
JOURNAL INFORMATION
==============================
NLM Title Abbreviation: ScientificWorldJournal
Journal ID: ScientificWorldJournal
NLM Journal ID: 101131163
Journal ID: TSWJ

The Scientific World Journal

ISSN: 2356-6140
EISSN: 1537-744X
Publisher: Wiley

ARTICLE INFORMATION
==============================
PMCID: PMC6084549
PMID: 12805726
DOI: 10.1100/tsw.2001.471
Article ID: 867652
Article version: 1
Subjects: Directions in Science

Vitamin A Prevents Inner Ear Defects in Mice with Congenital Homeobox Gene Deficiency

Pasqualetti Massimo
Rijli Filippo M.
Institut de Génétique et de Biologie Moléculaire et Cellulaire, CNRS/INSERM/ULP, Collège de France, C.U. de Strasbourg, France
Publication date: 2001
Electronic publication date: 2001 Dec 18
Volume: 1
First page: 916
Last page: 918
Copyright: Copyright © 2001 Massimo Pasqualetti and Filippo M. Rijli.
Copyright year: 2001
License: This is an open access article distributed under the Creative Commons Attribution License, which permits unrestricted use, distribution, and reproduction in any medium, provided the original work is properly cited.
License URL: https://creativecommons.org/licenses/by/3.0/

Keywords: Hox genes, brainstem, retinoic acid, cochlea, vestibular system, embryo development, Fgf signaling, knockout, rescue of congenital defect, maternal treatment

==============================
Directions in Science
*TheScientificWorld* (2001) 1, 916–918
ISSN 1532-2246; DOI 10.1100/tsw.2001.471

### Vitamin A Prevents Inner Ear Defects in Mice with Congenital Homeobox Gene Deficiency

Massimo Pasqualetti and Filippo M. Rijli

*Institut de Génétique et de Biologie Moléculaire et Cellulaire, CNRS/INSERM/ULP, Collège de France, BP 163 - 67404 Illkirch Cedex, C.U. de Strasbourg, France*

*Received November 19, 2001; Accepted November 28, 2001; Published December 18, 2001*

**KEY WORDS:** Hox genes, brainstem, retinoic acid, cochlea, vestibular system, embryo development, Fgf signaling, knockout, rescue of congenital defect, maternal treatment

**DOMAINS:** neuroscience, signaling, intercellular communication, development, growth and growth factors, embryology, nutrition, cell cycle (fate), differentiation and determination, sensation and perception, drug delivery, genetics, gene expression, molecular medicine

For the past 75 years, vitamin A and its biologically active metabolites, the retinoids, have been the object of intense study in biology and medicine. A large body of evidence demonstrates that these nutrients are essential for normal development and survival of vertebrate embryos, including mammals[1,2,3,4]. In fact, it has been known since the mid-1930s that vitamin A deficiency during pregnancy results in death of the fetus and congenital abnormalities. Similarly, excess dietary intake of vitamin A can also cause teratogenic responses. Among the main targets of both deficiency and excess retinoid-induced teratogenesis are the heart, limbs, craniofacial structures, central nervous system, and the inner ear. Specific malformations are induced in a stage- and dose-dependent manner. Thus, these studies indicate that precise levels and timing of action of vitamin A metabolites are required for normal patterning of embryonic structures. In addition, the discovery of the nuclear receptors for retinoic acid (RA) and other vitamin A derivatives provided a molecular basis to explain how distinct doses of these compounds elicit cell-specific responses via the direct transcriptional activation of a panel of target genes[5].

The homeobox genes of the *Hox* family are among the direct targets of retinoids in both normal and abnormal development[6,7,8,9,10]. These genes encode for transcription factors that operate at the top of a genetic hierarchy to control patterning of tissues and organs along the main axis of both invertebrate and vertebrate embryos. In recent years, the role of mammalian *Hox* genes has been investigated using gene targeting in the mouse. For instance, these studies established the involvement of *Hox* genes in the segmental patterning of the mammalian hindbrain[11]. *Hoxa1*, in particular, is required for the normal patterning of three hindbrain segments, namely the rhombomeres (r)4, 5, and 6[12,13]. The hindbrain is also known to provide inductive signalling for the development of the inner ear[14]. Inner ear structures are critical for the senses of hearing and balance and originate from the otocyst, which is formed adjacent to rhombomeres (r)5 and r6 at early developmental stages. It is therefore not surprising that mutations affecting patterning in the r4–r6 region can also cause profound malformations of inner

E-mail, F. Rijli:: rijli@igbmc.u-strasbg.fr ; http://www1-igbmc.u-strasbg.fr/~Rijli
Tel.: +33 3 8865 3460; Fax: + 33 3 8865 3201
©2001 with author.

ear structures. The *Hoxa1* targeted ablation, in particular, causes severe alterations of the otic capsule and membranous labyrinth morphogenesis[12].

As normal inner ear development requires both retinoid activity and *Hoxa1* function, we tested the hypothesis that a low dose of exogenous RA, although not sufficient to affect wild type fetus development, could bypass *Hoxa1* requirement and prevent the inner ear abnormalities of homozygous mutants. The results of this study were recently reported in *Nature Genetics*[15].

We showed that a single maternal oral administration of 5 mg $kg^{-1}$ RA was not teratogenic when given from 8.0 day post coitum (dpc) onward. Moreover, when mothers were administered this RA dose, the *Hoxa1* homozygous mutant fetuses were born with almost normal vestibular and cochlear structures. However, the rescue of inner ear defects was effective only if RA was provided within a certain window during pregnancy, namely between 8.0 and 8.75 dpc. This temporal window corresponds to the early stages of otocyst development and treatment outside this period did not rescue the inner ear phenotype. Thus, the RA response can compensate for the loss of *Hoxa1* function, but only when given at the opportune time. We then investigated the molecular response to the RA treatment and identified a molecular mechanism that may be involved in the recovery of inner ear development. *Kreisler* (*kr*), a transcription factor, and *Fgf3*, a signaling molecule, are expressed in r5 and r6 and have also been involved in inner ear patterning[16,17,18,19]. In *Hoxa1* homozygous mutant embryos, both *kr* and *Fgf3* expressions are significantly reduced, as well as that of the *Hoxa1* direct target *Hoxb1*[20]. In *Hoxa1* mutants treated at 8.0 dpc, the expression of these three genes was transiently restored. However, in embryos treated at 8.75 dpc only *Fgf3* was upregulated, yet the inner ear defects could be still rescued. As Fgf3 signaling from the hindbrain is thought to be essential for inner ear morphogenesis[21], our data strongly suggest that RA treatment of mutants, if timed correctly, can compensate for *Hoxa1* function in activating a *Fgf3*–dependent signaling pathway, and rescue inner ear development.

This study provides insight into the molecular mechanism through which *Hoxa1* may control inner ear development. It also provides further evidence[22] that prenatal supplementation of a nutrient, if given at the appropriate time, can be effective in reversing the developmental abnormalities resulting from a congenital genetic deficiency. It should be noted that, although a correlation exists between high retinoid levels and birth defects in humans, the precise dose at the limit of teratogenicity is still controversial. The U.S. and European Teratology Societies have recommended that overall vitamin A intake during pregnancy should not exceed 8,000 to 10,000 IU/day, to avoid any risk of developmental toxicity[23]. However, consumption during pregnancy of supplemental vitamin A at levels present in multivitamin preparations does not appear to increase the risk for birth defects[1]. It is therefore tempting to speculate that, although caution is still needed in extrapolating results from the mouse to potential treatment in humans, low retinoid levels from dietary intake during gestation might, under some circumstances, have therapeutic effects.

### REFERENCES

1. Ross, S.A., McCaffery, P.J., Drager, U.C., and De Luca, L.M. (2000) Retinoids in embryonal development. *Physiol. Rev.* **80**, 1021–1054.
2. Zile, M.H. (2001) Function of vitamin A in vertebrate embryonic development. *J. Nutr.* **131**, 705–708.
3. Niederreither, K., Subbarayan, V., Dolle, P. , and Chambon, P. (1999) Embryonic retinoic acid synthesis is essential for early mouse post-implantation development. *Nat. Genet.* **21**, 444–448.
4. Collins, M.D. and Mao, G.E. Teratology of retinoids. (1999) *Annu. Rev. Pharmacol. Toxicol*. **39**, 399–430.
5. Chambon, P. (1996) A decade of molecular biology of retinoic acid receptors. *FASEB J.* **10**, 940–954.
6. Dupe, V., Davenne, M., Brocard, J., Dolle, P., Mark, M., Dierich, A., Chambon, P., and Rijli, F.M. (1997) In vivo functional analysis of the Hoxa-1 3' retinoic acid response element (3'RARE). *Development* **124**, 399–410.

7. Simeone, A., Avantaggiato, V., Moroni, M.C., Mavilio, F., Arra, C., Cotelli, F., Nigro, V., and Acampora, D. (1995) Retinoic acid induces stage-specific antero-posterior transformation of rostral central nervous system. *Mech. Dev.* **51**, 83–98.
8. Conlon, R.A. and Rossant, J. (1992) Exogenous retinoic acid rapidly induces anterior ectopic expression of murine Hox-2 genes in vivo. *Development* **116**, 357–368.
9. Kessel, M. and Gruss, P. (1991) Homeotic transformations of murine vertebrae and concomitant alteration of Hox codes induced by retinoic acid. *Cell* **67**, 89–104.
10. Marshall, H., Morrison, A., Studer, M., Popperl, H., and Krumlauf, R. (1996) Retinoids and Hox genes. *FASEB J.* **10**, 969–978.
11. Pasqualetti, M. and Rijli, F.M. (2001) Homeobox genes and brainstem developmental disorders: learning from knockout mice. *Curr. Opinion Neurol.* **14**, 177–184.
12. Mark, M., Lufkin, T., Vonesch, J., Ruberte, E., Olivo, J., Dolle, P., Gorry, P., Lumsden, A., and Chambon, P. (1993) Two rhombomeres are altered in Hoxa-1 mutant mice. *Development* **119**, 319–338.
13. Carpenter, E.M., Goddard, J.M., Chisaka, O., Manley, N.R., and Capecchi, M.R. (1993) Loss of Hox-A1 (Hox-1.6) function results in the reorganization of the murine hindbrain. *Development* **118**, 1063–1075.
14. Brigande, J.V., Kiernan, A.E., Gao, X., Iten, L.E., and Fekete, D.M. (2000) Molecular genetics of pattern formation in the inner ear: do compartment boundaries play a role? *Proc. Natl. Acad. Sci. U. S. A.* **97**, 11700–11706.
15. Pasqualetti, M., Neun, R., Davenne, M., and Rijli, F.M. (2001) Retinoic acid rescues inner ear defects in *Hoxa1* deficient mice. *Nat. Genet.* **29**, 34–39.
16. McKay, I.J., Muchamore, I., Krumlauf, R., Maden, M., Lumsden, A., and Lewis, J. (1994) The kreisler mouse: a hindbrain segmentation mutant that lacks two rhombomeres. *Development* **120**, 2199–2211.
17. Frohman, M.A., Martin, G.R., Cordes, S.P., Halamek, L.P., and Barsh, G.S. (1993) Altered rhombomere-specific gene expression and hyoid bone differentiation in the mouse segmentation mutant, kreisler (kr). *Development* **117**, 925–936.
18. McKay, I.J., Lewis, J., and Lumsden, A. (1996) The role of FGF-3 in early inner ear development: an analysis in normal and kreisler mutant mice. *Dev. Biol.* **174**, 370–378.
19. Mansour, S.L., Goddard, J.M., and Capecchi, M.R. (1993) Mice homozygous for a targeted disruption of the proto-oncogene int-2 have developmental defects in the tail and inner ear. *Development* **117**, 13–28.
20. Studer, M., Gavalas, A., Marshall, H., Ariza-McNaughton, L., Rijli, F.M., Chambon, P., and Krumlauf, R. (1998) Genetic interactions between Hoxa1 and Hoxb1 reveal new roles in regulation of early hindbrain patterning. *Development* **125**, 1025–1036.
21. Pirvola, U., Spencer-Dene, B., Xing-Qun, L., Kettunen, P., Thesleff, I., Fritzsch, B., Dickson, C., and Ylikoski, J. (2000) FGF/FGFR-2(IIIb) signaling is essential for inner ear morphogenesis. *J. Neurosci.* **20**, 6125–6134.
22. Chen, W.H., Morriss-Kay, G.M., and Copp, A.J. (1995) Genesis and prevention of spinal neural tube defects in the curly tail mutant mouse: involvement of retinoic acid and its nuclear receptors RAR-beta and RAR-gamma. *Development* **121**, 681–691.
23. Senior, K. (2001) Vitamin A and its impact on human medicine. *The Lancet* **358**, 1072.

**This article should be referenced as follows:**

Pasqualetti, M. and Rijli, F.M. (2001) Vitamin A prevents inner ear defects in mice with congenital homeobox gene deficiency. *TheScientificWorld* 1, 916–918.
